# The Role of Oxidative Stress and Hormones in Controlling Obesity

**DOI:** 10.3389/fendo.2019.00540

**Published:** 2019-08-13

**Authors:** Marina Di Domenico, Federica Pinto, Lucio Quagliuolo, Maria Contaldo, Giuliana Settembre, Antonio Romano, Mario Coppola, Kenan Ferati, Arbëresha Bexheti-Ferati, Antonella Sciarra, Giovanni Francesco Nicoletti, Giuseppe Andrea Ferraro, Mariarosaria Boccellino

**Affiliations:** ^1^Department of Precision Medicine, University of Campania Luigi Vanvitelli, Naples, Italy; ^2^Department of Biology, College of Science and Technology, Temple University, Philadelphia, PA, United States; ^3^Multidisciplinary Department of Medical-Surgical and Dental Specialties, University of Campania Luigi Vanvitelli, Naples, Italy; ^4^Faculty of Medicine, University of Tetovo, Tetovo, Macedonia; ^5^Department of Translational Medicad Sciences, University of Campania Luigi Vanvitelli, Naples, Italy; ^6^Plastic Surgery Unit, Multidisciplinary Department of Medical-Surgical and Dental Specialties, University of Campania Luigi Vanvitelli, Naples, Italy

**Keywords:** obesity, oxidative stress, thyroid, gut hormones, microbiota, wound healing

## Abstract

The accumulation of adipose tissue in the body occurs because the energy introduced with food and drink exceeds that expense, but to understand why this imbalance is established and why it is maintained over time, it is important to consider the main causes and risk factors of excess weight. In this review, we will refer to the main factors linked to obesity, starting from oxidative stress to hormonal factors including the role of obesity in breast cancer. Among the many hypotheses formulated on the etiopathology of obesity, a key role can be attributed to the relationship between stress oxidative and intestinal microbiota. Multiple evidences tend to show that genetic, epigenetic, and lifestyle factors contribute to determine in the obese an imbalance of the redox balance correlated with the alteration of the intestinal microbial flora. Obesity acts negatively on the wound healing, in fact several studies indicate morbid obesity significantly increased the risk of a post-operative wound complication and infection. Currently, in the treatment of obesity, medical interventions are aimed not only at modifying caloric intake, but also to modulate and improve the composition of diet with the aim of rebalancing the microbiota-redox state axis.

## Introduction

### Obesity

In the last few years the changes in diet and lifestyle resulted in an prevalence of overweight and obesity. Obesity rates have increase in the last several decades; more than 30% of the US population is now classified as obese (1, 2). The prevalence of these individuals varies from country to country but in developed countries, most of the population are affected. Just think that in America about a third of population are obese with a body mass index > 30 kg/m^2^ (3). The variation from country to country implies that environmental factors are the major determinant of disease prevalence. Obesity is thought to be the second most preventable cause of death after smoking; moreover Roland Sturm in his study suggests that the health care costs of obesity exceed those of smoking (4). In particular, he compared the effects of obesity, overweight, smoking, and problem drinking on health care. Obesity is associated with a 36% increase in inpatient and outpatient spending and a 77% increase in medications, compared with a 21% increase in inpatient and outpatient spending and a 28% increase in medications for smokers and smaller effects for problem drinkers. Obesity is also increasing in children. The consumption of sweetened beverages is associated with childhood obesity but also because they spend a considerable part of their lives watching television (5, 6).

Obesity is a complex disease associated with disturbances in lipid and glucose metabolism, chronic inflammation, oxidative stress, and an increased risk of several diseases, most notably cardiovascular diseases, diabetes, and cancers (7, 8). In obesity, the skin is one of the main organs to be affected through a complex interaction of hormones, adipocytokines, and mechanical factors. The most common skin diseases associated with obesity are keratosis pilaris, cellulite, candidiasis/dermatophytosis, psoriasis, and Lichen planus (9, 10). Obesity is an important risk factor for several malignancies. In fact, higher body mass index (BMI) increases the incidence of many types of cancer. The role of obesity in head and neck squamous cell carcinoma (HNSCC) is not well-defined. However, obesity may be applied to predict prognosis of oral squamous cell carcinoma (OSCC) patients (11, 12). The expression of adhesion molecules such as cadherins during the epithelial mesenchimal transition (EMT) can improve OSCC prognosis and therapy (13, 14).

**Figure 1:**
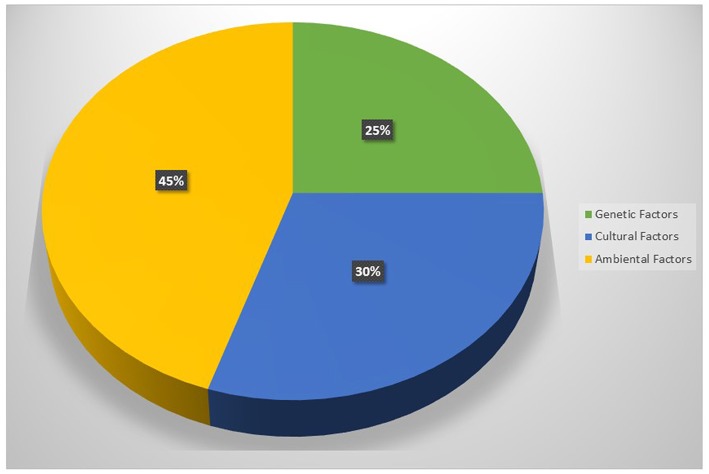
The main causes of obesity.

Adipose tissue is no longer viewed as a passive source of free fatty acids (FFA) but as an active endocrine and paracrine organ secreting an ever-increasing number of mediators named adipokines. These secreted proteins include tumor necrosis factor (TNF)-α, resistin, IL-6, acylation-stimulating protein (ASP), angiotensinogen (AGT), plasminogen activator inhibitor-1 (PAI-1), leptin, and adiponectin. They participate in diverse metabolic processes including food intake, fat metabolism, feeding behavior, haemostasis, vascular tone, energy balance, and insulin sensitivity regulation of energy balance (15–18). Leptin and adiponectin exert a beneficial effect on energy balance, insulin action, and vasculature while excessive production of fatty acids (FA) and TNF-α, IL-6, and resistin is deleterious because they might deteriorate insulin action. Angiotensinogen and PAI-1 are likely to participate in the vascular complications linked to obesity (19). In obese individuals, excessive production of ASP, TNF-α, IL-6, or resistin deteriorates insulin action in muscles and/or in liver, while increased angiotensinogen and PAI-1 secretion favors hypertension and impaired fibrinolysis (20–22). Leptin is a 16-kDa protein synthesized mainly in adipose tissue in fact adipocytes are the most important source of leptin (23). The primary role of leptin is the control of appetite, mutations in the leptin gene, or leptin receptor gene develop obesity (19, 24). Several studies indicate a direct role of leptin in lipid metabolism mediated both through central and peripheral actions of it (Figure 1). In rodent models, central administration of leptin increased resting metabolic rates, resulting in reduced triglycerides content in both adipose and non-adipose tissues, as well as reduced plasma free FA and triglycerides levels. Leptin may also have autocrine or paracrine effects on adipocyte fat metabolism. The incubation of mouse adipocytes with leptin stimulates lipolysis of intracellular triglycerides (TG), and this effect was not seen in in diabetes mice lacking leptin receptors (25, 26).

## The Role of Oxidative Stress

Experimental evidences suggest that the sources of oxidative stress in obesity are different as hyperglycemia, hyperleptinemia, inadequate antioxidant defenses, increased muscle lipid levels, increased muscle activity, increased free radical formation rates, alteration of mitochondrial function, endothelial dysfunction, and chronic inflammation (27, 28). Many studies have suggested that natural compounds as phytochemicals in fruits and vegetables can be important modulators in terms of the risks associated with obesity. In particular, Martineau and Sakaida have demonstrated the anti-diabetic and anti-hypertensive properties of the blueberry *in vitro* (29, 30). In addition, in obese Otsuka Long-Evans Tokushima Fatty (OLETF) rat, a rat model that develops a syndrome with multiple metabolic and hormonal disorders that shares many features with human obesity, it has been shown that the blueberry leaves extracts, especially flavonol glycoside and proanthocyanidin, had a hypolipidemic effect on OLETF rats, and suggest that an infusion of blueberry leaves extracts could be useful as a dietary hypolipidemic component (31).

Oxidative stress and inflammation, which occur in obesity, can induce DNA damage and inhibit DNA repair mechanisms that lead to an increase in mutation frequency and can alter gene expression. DNA damage associated with obesity can promote cancer growth by favoring cancer cell proliferation and migration, and resistance to apoptosis (32–37).

**Figure 2:**
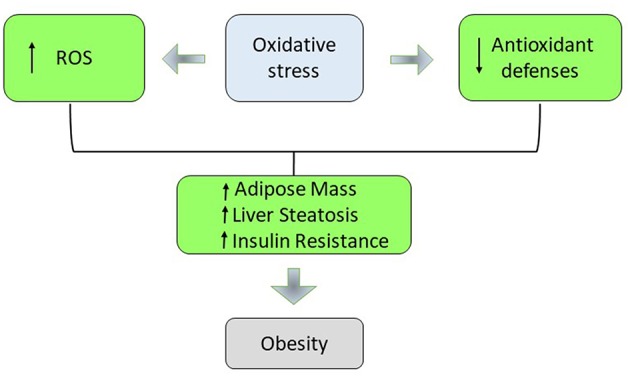
The role of oxidative stress in obesity.

In obesity, during the hyperglycaemia, the intracellular glucose overload increases the glycolysis and the Krebs cycle, generating an increase of NADH and FADH_2_ that lead to the end of the oxidative phosphorylation to the superoxide production. In fact, augmented respiration may not be bioenergetically efficient due to leaking mitochondria and thereby promote excessive hepatic oxidative stress, challenging hepatocellular anti-oxidant defense mechanisms (38–40). In non-alcoholic fatty liver (NAFL) augmented hepatic oxidative stress (H_2_O_2_ and lipid peroxides) and oxidative DNA damage (8-OH-deoxyguanosine) was balanced by reduced antioxidant defense capacity and increased inflammatory response. If the antioxidant defense mechanism fails to counteract oxidative stress, mitochondrial functionality decreases developing hepatic insulin resistance, systemic inflammation, and NAFLD progression to steatohepatitis (NASH). This suggests an adaptation of hepatic mitochondria in obese humans without NASH (41). The increase in plasma FFA promotes the generation of O^2−^ in the mitochondrial electron transport chain by inhibiting the translocation of adenine nucleotides. Conjugated fatty acids are susceptible to oxidation, stimulate the formation of radicals, and enhance the accumulation of oxidative by-products (42). Various studies have suggested an association between levels of different markers of systemic oxidative stress and the accumulation of fat (43). There is an important role for adipose tissue in the production of ROS. The increase in ROS in adipose tissue has been associated with increased expression of NADPH oxidase and reduced expression of anti-oxidant enzymes such as SOD and catalase (44). The removal of free radicals occur through enzymic and non-enzymic antioxidants but an increase in weight can reduce the antioxidant capacity of plasma (45). The weight loss and BMI reduction might induce an increase of antioxidant enzymes activities in the obese individuals. Moreover, the activity of these enzymes is influenced by the daily intake of antioxidant vitamins (46, 47). In the obese individuals, inadequate concentrations of vitamins and minerals cause the observed impaired antioxidant defense (48, 49). In fact an increase in BMI has been found to be related to low levels of carotenoids, vitamin C, and vitamin E. Adequate intracellular antioxidant defenses are necessary to maintain the antioxidant–pro-oxidant balance in tissues. Oxidative stress also plays an important role not only in biochemistry and cell biology but also in the nutritional sciences, environmental medicine, and molecular knowledge-based redox medicine. Therefore, research on this topic is relevant for maintaining health condition, for using drugs and for a better understanding of various diseases. Oxidative stress is closely associated with pathological mechanisms and symptoms of urinary bladder dysfunction. In particular, partial bladder outlet obstruction (PBOO) causes pathological changes in bladder tissues through induction of oxidative stress (50, 51). Low levels of ROS are considered essential for neuronal development and function, while excessive are hazardous. Indeed, the brain, with its high energy demand, and weak antioxidant capacity becomes an easy target of excessive oxidative stress. Thus, ROS accumulation is a cellular threat that, if it bypasses counteracting mechanisms, can cause significant neuronal damage (52). The rapid development and use of nanotechnology products has led to their ever wider use in the biomedical field. Nanoparticles are fundamental tools for medicine and biology, as they can be used for biomedical applications from diagnosis to therapy. There are many experimental researches for the production and characterization of biocompatible nanoparticles that can become efficient carriers to be used in drug delivery in different types of diseases. Biological systems and nanoparticles can be used to study cell toxicity, apoptosis in human mucosa, in particular nanoparticle biomolecular corona can be correlated to physiological and pathological conditions (53–55). Deficiencies in vitamins and minerals can also contribute to the development of an impaired antioxidant defense in the pathogenesis of obesity (56, 57). Kimmons et al. have examined the association between BMI and micronutrient levels. In particular, they have measured nutritional biomarker levels in the serum such as alpha-carotene, beta-carotene, lycopene, vitamin E, vitamin C, vitamin A, vitamin D, folate, and vitamin B12. Overweight and obese adults had higher odds of low levels for a number of nutrients than normal-weight adults. Odds of being low in multiple micronutrients was most common among overweight and obese premenopausal women (58). The chronic low-grade state of inflammation in obesity is another important source of oxidative stress. TNF-α, IL-6, IL-8, and IL-1 are the most well-known mediators of the early inflammatory response that are over-expressed in obesity and increase the activities of Nicotinamide Adenine Dinucleotide Phosphate (NADPH) oxidases (NOXs) and the production of superoxide anion (59–61). Adipocytes are the most important source of leptin and plasma leptin concentrations are associated with the amount of adipose tissue. Leptin influences appetite, in fact its mutation or mutation in its receptor leads to obese subjects (20, 24). Leptin plays an important role in obesity-induced oxidative stress. It activates NOX and induces the production of reactive intermediates such as H_2_O_2_ and hydroxyl radical (Figure 2). In a rodent model, leptin injection caused higher levels of plasma and urinary lipid hydroperoxide, malondialdehyde (MDA), isoprostane, and protein carbonyl content. In addition, leptin also stimulates the production of proinflammatory cytokines and reduces the activity of the cellular antioxidant paranoxase-1 (PON-1) (62, 63). The activation of metabolic pathways generate increased of free radicals and electron transport chain activity. This leads to exacerbate the cellular respiration rate and oxygen uptake in muscle tissue during physical activities. Obese individuals are also mechanically less efficient during exercise and this insufficiency contributes to the increased energy expenditure for a given exercise load. An increase in mitochondrial respiration for energy production imply higher levels of lipid hydroperoxide in obese people (64, 65). Obesity is associated with higher concentrations of angiotensin II that promote oxidative stress in vasculature through several mechanisms including activation of NOX, formation of O2^−^, and production of H_2_O_2_ (66). Excessive energy substrate causes mitochondrial dysfunction, which has been linked to the dysregulated secretion of adipokines, defects in fatty acid oxidation, increased production of ROS, and alteration of glucose homeostasis (67). Moreover, during the adipocyte differentiation process, the mitochondrial biogenesis and activity increase rapidly. These organelles play a central role in ATP production, energy expenditure, and disposal of ROS. When there is an excess of electrons as in obesity, a reduction of oxygen occurs resulting in the formation of potentially toxic-free radicals (68, 69). Diet is another possible contributing factor in the generation of ROS during obesity. In fact, consumption of a high-fat diet may alter oxygen metabolism. Moreover, obese individuals possess a lower dietary intake of protective phytochemicals rich in antioxidants such as β-carotene, vitamin E, and C, zinc, selenium, whereby it generate an inadequate antioxidant defense (70).

## Thyroid Hormones

**Figure 3:**
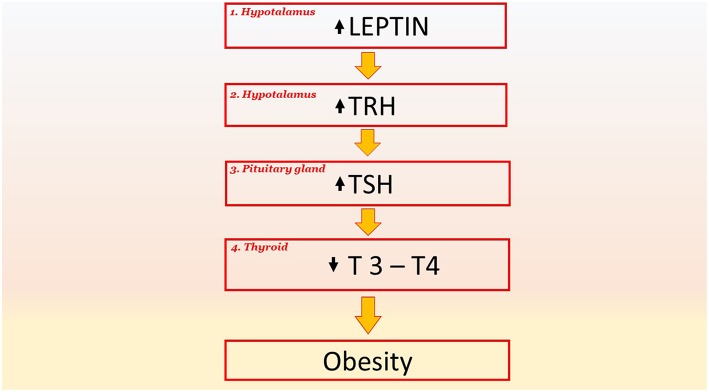
The key role of TSH signaling and their downstream effectors in pathological phenomena and obesity. Leptin stimulates the production of TRH in the hypothalamus (point 1). An increase in TRH induces an increase in TSH in the pituitary gland (point 2); this increase results in a decrease in the hormones T3 and T4 in the thyroid (point 3). The reduced production of T3 and T4 reduces the basal metabolism by 40% and this favors obesity. TRH, Thyrotropin-releasing hormone; TSH, Thyroid-stimulating hormone; T4, thyroxine; T3, triiodothyronine.

Thyroid dysfunction is highly widespread in the population. National data suggest that hypothyroidism is present in 4.6% of the US, and hyperthyroidism in 1.3%. Thyroid dysfunction is very prevalent in obese subjects, in fact it was observed that among about 800 obese patients seen for bariatric surgery evaluation about 20% of this had elevated serum TSH (71). Recent clinical studies show the correlation between thyroid treatments and weight change (Figure 3). Patients with hypothyroidism have a persistent weight while following a diet, whereas patients with hyperthyroidism frequently present a weight loss. It was observed that weight decreases after treatment for hypothyroidism. Different studies describe weight change after the start of treatment for thyroid dysfunction, in particular, it was observed that more than 50% of patients with hypothyroidism lost weight after the start of levothyroxine therapy (L-T4) and 2 years following initiation of treatment, they had regained their baseline weight. Another study looked at the effects of liothyronine (L-T3) compared to treatment with L-T4. Treatment with L-T3 (19 weeks) resulted in significant weight loss (1.8 kg on average) and a significant decrease in fat mass (72). The close relationship between body weight and hormonal function is also demonstrated in a retrospective study, in which 120 patients that have achieved euthyroidism with thyroid hormone therapy 1 year following total thyroidectomy was compared with treated hypothyroid individuals who did not underwent thyroidectomy. After a year, the thyroidectomised patients had experienced significantly more weight gain (3.1 vs. 2.2 kg, *p* = 0.004) than individuals who did not undergo thyroidectomy (73–75). The state of the thyroid can affect the distribution and quantity of the adipose tissue. In several studies it has been observed that the amount of subcutaneous fat and subcutaneous adipose tissue is inversely proportional with free thyroxine (FT4) levels whilst the increase in TSH is correlated with the thickness of subcutaneous fat (76). Indeed it was demonstrated a positive correlation between triiodothyronine (T3) and body weight. The relationship between the state of the thyroid and obesity is bidirectional: not only does thyroid function affect the state of obesity but obesity also affects thyroid function. The reason for the elevations in both TSH and T3 is attributable to leptin (77, 78). During caloric restriction there is a reduction in the concentration of leptin, thyroid hormones (T3) and catecholamine. This allows a metabolic adaptation that persists, hinders further weight loss, and pre-disposes some subjects to regain weight. In particular, it has been shown that in response to caloric restriction the leptin concentration decreases within 24 h, regardless of circadian changes. The hypothalamus responds by decreasing the levels of thyroid hormones and sympathetic activation and skeletal muscle by increasing the efficiency of the mitochondria (79, 80). It can therefore be concluded that both serum TSH and T3 are dramatically increased in obese and it is caused, at least in part, by an impaired leptin production (81–83).

## Gut Hormones

**Figure 4:**
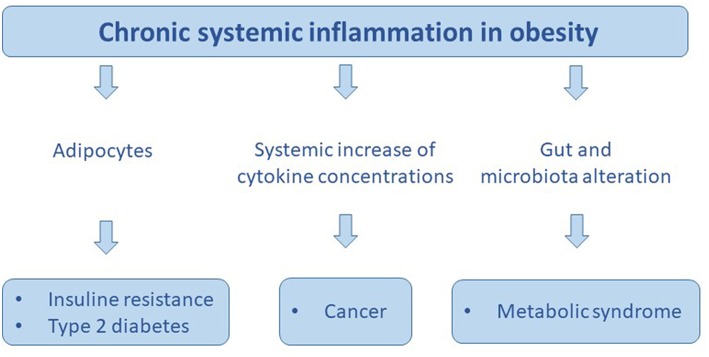
The chronic sistemic inflammation in obesity promotes: insuline resistance, type 2 diabetes, cancer, and metabolic syndrome.

The gastrointestinal tract, that is largest endocrine organ and its secretion of important gut hormones such as ghrelin, cholecystokinin (CCK), peptide YY (PYY), and glucagon like peptide-1 (GLP-1) plays a central role in metabolism and in maintaining of the baseline weight. This hormones regulate metabolism coordinating appetite, digestion, absorption, and nutrient disposal. The main role of intestinal hormones is to promote the absorption of nutrients but an imbalance of these hormones causes serious damage to health (84, 85). Gastrointestinal hormones play a central role in control of insulin secretion, for this reason they are essential for body weight and blood glucose control. In fact, bariatric surgery is currently the most successful treatment for the treatment of type 2 diabetes and obesity. This data suggests the close relationship between intestinal hormones and obesity (86). Gut hormones concentration varies between individuals and this depends on physiological and pathological conditions of individual. Metabolic changes, increased adiposity, altered diet are all factors that affect the production of these hormones with serious damage to health in obese subjects (Figure 4). The incretins are hormones produced by some cells in the intestine and released into the blood during a meal. The most important incretins are glucagon-like peptide 1 (GLP-1) and glucose-dependent insulinotropic polypeptide (GIP), both of which have the effect of glucose-dependent promotion of insulin release by pancreatic beta cells. Furthermore, GLP-1, combined with the increase in insulin levels, reduces glucagon secretion by the alpha cells of the pancreas. Therefore, during a meal, the increase in incretin levels helps, in a glucose-dependent manner, to release a greater share of insulin and to produce less glucagon, obtaining a better control of glycemia, the so-called “incretinic effect.” In healthy individuals “the incretinic effect” is interrupted after 2–5 min by an enzyme: DPP-IV or Di-Peptidyl-Peptidase IV (87, 88). A reduction in GLP1 secretion in the gastrointestinal tract was observed, in obese subjects with diabetes mellitus. The somministration of GLP-1 is efficacious in improving glycaemia in T2DM patients (87–89). A limit to the therapeutic use of GLP-1 is its short halflife, due to rapid degradation by dipeptidyl peptidase 4 (DPP4). To avoid this problem, DPP4-resistant GLP-1 analogs have been developed that possess enhanced half-lives, making treatment efficient. GLP-1 analogs are only licensed for T2DM, but some researchers have tested their use for the treatment of obesity without diabetes. It was showed that liraglutide (an analogous of GLP-1 in commerce), at doses of up to 3.0 mg daily, was able to induce weight losses of up to 7.2 kg on average (87). In obese subjects also the concentration of the Peptide YY is altered. Peptide YY (PYY) is expressed by L-cells of the intestinal mucosa of the ileum and colon and belongs to the family of neuropeptide Y (NPY) along with pancreatic polypeptide (PP). It inhibits intestinal contractions, pancreatic and gastric secretions and reduces appetite (90). The level of PYY in the blood increases after a meal and remains high for a few hours suggesting its role as a satiety factor (91, 92). It arrives, through the bloodstream, to the arcuate nucleus of the hypothalamus where it acts on the orissigenic neurons by inhibiting the release of NPY and reducing hunger (93, 94). A study in volunteers suggests that PYY3–36 may increase insulin secretion in response to a meal, with an increase in glucose levels, suggesting that with PYY3–36 insulin sensitivity is reduced. PYY3–36 analogs are in experimentation for obesity and Diabetes mellitus type 2 treatments (95). In humans, fasting PYY and post-glucose GLP1 concentrations are inversely related to BMI, suggesting that low levels of PYY and GLP1 may pre-dispose to obesity (96).

### Microbiota

The intestinal microbiota, the complex and dynamic population of micro-organisms that inhabits intestine, plays a key role in the absorption of nutrients, in the accumulation of energy and in the regulation of various metabolic pathways, able to influence body weight (97).

Saliva plays an important role in determining the composition and activity of the oral microbiota. The molecules coming from saliva form a conditioning film on oral surfaces, thus providing receptors for bacterial attack (98, 99).

It has been observed that the microorganisms that colonize the gastrointestinal tract are not only almost inert hosts but are active protagonists of interactions between the gastrointestinal tract and the neuro-immuno-endocrine system (100, 101). The colonic microbiota produces several metabolites, many of these modulate the activity of the surrounding host enteroendocrine cells population. Human enteroendocrine cell responding to infection to *chlamydia trachomatis* are detected in gastrointestinal tract (102, 103). The microbiota derived luminal metabolome is the sum of many bacterial species and depends on the quantity and nature of the bacterial population, as well as the composition of the nutrients. The inter-relationship between IgAs and the microbiota is an open question. Even though recent publications have shown that IgAs can participate to microbiota diversification and that the microbiota can drive IgA production in a T-cell-independent manner, it is still unclear how the two are interacting and influencing each other (104). Microbioma metabolites modulate several of enteroendocrine cells population pathways, including expression profiles, hormone biosynthesis, and stimulus–secretion coupling pathways. In primary cultures it was shown that the expression of the PYY gene was induced by butyrate, a short chain fatty acid (SCFA) produced by bacterial fermentation of the fibers, via a pathway linked to the inhibition of histone deacetylase (HDAC). Thus, the consumption of fibers improve satiety in humans (105–108). Studies conducted on mouse models have shown that the microbiota of obese mice promotes the extraction of additional calories from the diet, favoring weight gain. Furthermore, by transplanting the intestinal microbiota of obese mice or skinny mice into lean, aseptic mice, mice that had received the microbiota from the obese were able to extract more calories from the foods showing a significantly greater accumulation of fat than the mice that received the microbiota from lean mice (109). The alteration in the intestinal microbial composition of humans is associated with obesity; in obese subjects the relationship between the two bacterial groups that dominate the human gut, Firmicutes, and Bacteroidetes, with a relative abundance of the first to the detriment of the latter, changes (110, 111). The differences in the extraction of calories from substances ingested with food can be largely dependent on the composition of the intestinal microbiota and, at the same time, weight loss is able to restore the normal intestinal microbial composition, confirming the link between microbiota and obesity (112, 113). Currently it is increasingly possible, to use the manipulation of the intestinal microbiota as a therapeutic strategy to regulate the energy balance in obese, diabetic or diagnosed with metabolic syndrome (114). In fact, several studies have shown the benefit of supplementation with probiotics in weight loss interventions; for example, low-calorie diets associated with the consumption of probiotics favor a more substantial weight loss and a reduction of the upper visceral fat, compared to diet alone. Furthermore, the use of probiotics allows faster weight loss in patients undergoing bariatric surgery (115). The usage of pre-biotics, such as fructan (prebiotics obtained from chicory root) or arabinoxylans (obtained from bran) also would reduce fat mass and inflammation, favoring the colonization of “good bacteria” inside the microbiota, thus strengthening the intestinal defenses (116).

## Breast Cancer

Breast cancer (BCa) is the most common cancer among women both in developed and in developing countries (117). In young women, namely 40 years old or younger, hormone responsive tumors are associated with epigenetic and hereditary factors (118, 119). Deregulation of epigenetic mechanisms may lead to human diseases including cancer and several dietary compounds such as catechins, curcumin, resveratrol exhibit potent anti-tumor activities through the reversion of epigenetic alterations (120–123). Activation-inactivation of hormone binding sites depends by the phosphorylation of a calmodulin stimulated tyrosine kinase (124–126). Breast cancer in young women are almost 7% of the diagnosed women in Western populations (127). Considering that most of them have tumors positive to estrogen receptors, it is important to underline that in premenopausal period the ovaries represent the source of estrogen, while in postmenopausal period the main source is the adipose tissue. Interplay steroids receptors and neoplastic progression in sarcoma and adenocarcinoma tumors is known in hormone responsive signaling (128, 129). The excessive storage of lipids that occurs in obesity leads to important changes in adipose tissue, as adipocyte cell death and the recruitment of macrophages, with a consequent chronic low-grade inflammation and the activation of NFkB, also in the breast fat; as well-known, it may have important effects for tumor, both in its development and in its progression. This process is associated with severe alterations in growth and proliferation signaling, e.g., mitogen-activated protein kinase (MAPK) signaling, nuclear factor κ B (NFκB) signaling, by deregulation of signal transduction and protein-protein interactions (PPI) (130–132). Adipose tissue plays a central role in the alteration of tumor microenvironment releasing FFA, inflammatory cytokines, and adipose stem cells that can contribute to both to the remodeling of tumor microenvironment. All molecules in concert cooperate to creating the premetastaticed niches. Citokines and growth factors released in the neoplastic site are a biomarker signature and play a central role in the formulation of early tumor diagnosis and therapy. A treatment approach for invasive breast carcinoma and other cancer could be based on electroporation (133–137). As a matter of fact, obesity and metabolic syndrome are universally recognized as risk factors for a number of cancers, increasing in particular the risk of postmenopausal ER-positive BCa by over 50% (138). The mechanism that can explain this connection is still not completely clear, though it seems that the increase of serum insulin and insulin growth factors, as well as the higher levels of estrogens and the already mentioned inflammatory factors, may be the most common factors, identified as cancer signature for the clinical outcome of neoplastic patients. Recently, microRNAs (miRNAs) have shown promise as new biomarkers for many cancers, including metastatic breast cancer (139–141). Insulin-like growth factor-1 receptor (IGF-IR) activation is associated with the invasion and metastases in BCa and it has a role with the estrogen receptor in promoting tumor growth (142, 143). Vascular endothelial growth factor (VEGF) in concert with steroid receptors can be used for differential diagnosis of benign and malignant origin (144, 145). Moreover, hypercholesterolemia, that often affects obese women, may itself represent an independent risk factor. In fact, the BCa incidence, as well as its recurrence, is lower for patients that assume statins to reduce cholesterol level (146). A recent study pointed out fatty acids, and in particular low basal docosahexaenoic acid (DHA) levels as an important factor in increasing BCa risk and even in affecting prognosis and response to treatments, though the studies about an anticancer effect of DHA are often controversial (147). Liquid biopsy is used in breast and other cancer diagnosis. Furthermore, circulating tumor cells (CTC) plasma count can be assessed to determine the stage and prognosis of epithelial cancers (148). Over-expression of matrix metalloproteinases (MMPs) and tissue inhibitor of metalloproteinase are associated with the relapse of neoplatic disease, metastasis, shorter overall survival in breast, and other tumors MMPs/TIMPs ratio could be useful in the follow-up of these patients (149). In general, about 35% of cancer cases is influenced by nutrition (150) and in particular, several studies showed that gaining weight during postmenopausal life increases the risk of developing breast cancer, while a weight loss is associated with a reduced risk. In fact, several studies show that the state of low-grade chronic inflammation due to obesity may be reversible through a controlled diet and exercise, though exercise alone cannot do it (131). A 12 months weight loss diet, associated with physical exercise in postmenopausal women resulted in the reduction of several biomarkers of inflammation, suggesting that this effect may be crucial to reduce the risk of BCa as well as of other cancers in obese postmenopausal women (151). A valid example comes from a review of 33 intervention studies in which 1 kg of weight loss corresponds to 0.13 mg/l reduction of C-reactive protein (152). Diet and exercise are obviously modifiable factors, which may influence both the risk and recurrence of BCa. However, several studies such as Feigelson's have shown that the impact of weight loss on breast cancer risk has been difficult to quantify (153). The risk is strictly connected to serum sex hormones namely estrogens and androgens: it has been shown that the risk is up to two-fold in postmenopausal women with higher endogenous sex hormones levels. Weight loss induced by both diet and exercise has been demonstrated to have an important favorable effect on some of these hormones (154). Moreover, even during the follow up of BCa treatment, a diet rich in fruits and vegetables and poor in fat intake may reduce the risk of relapse after 5 years (155). Since 1999, Saxe et al. (156) analyzed the behavior of 149 women after BCa diagnosis, and showed that fat intake (of course together with other factors, for example lymph node positive status and tumor stage) was associated with an increased risk of death. In fact, fat intake promotes oxidative stress, and inflammatory signaling, that as already said, are involved in BCa recurrence (127). An important meta-analysis of prospective studies was conducted by Bauer et al. in (157), and they found a modest inverse association between circulating 25-hydroxyvitamin D and BCa risk as concerning postmenopausal women, while apparently there was no association for younger women (157), Granulocyte macrophage colony-stimulating factor (GM-CSF) and matrix metalloproteinase 9 (MMP-9) play a central role in breast cancer and other tumors (158–163). They are produced by mesenchymal progenitor cells or adipose stem cells (ASC) and act together promoting the development and spread of cancer. In obese mice, the levels of GM-CSF increase and this triggers a mechanism that causes them to produce further GM-CSF and MMP-9 (164). Together the two molecules make the breast cancer cells more aggressive, more prone to give rise to metastases and more active in turning off the response of the immune system against the tumor itself (164). This data suggests that the obese population has a higher risk of developing cancer due to obesity-associated adipose inflammation, which increase adipose secretion of pro-inflammatory factors like MMP and alter the tumor microenvironment (165, 166). A healthy diet associated with a correct prevention and a sports practice reduces cancer risk, attenuating adipose-related inflammatory mechanisms than can regulate cancer growth.

## Obesity and Wound Healing

Obesity acts negatively on the wound healing both for local factors, as increased traction of the wound edges, fat necrosis, ease of bleeding, and infections, and for general factors possibly associated such as diabetes arteriosclerosis hypertension. Wound healing is a complex process that involves local, regional, and systemic response elements. Surprisingly, in spite of numerous reports of post-surgical complications including wound infection, delayed closure, dehiscence, hematoma, and seroma formation, pressure ulcers, and venous ulcers, little research has been conducted to investigate the mechanisms mediating obesity-related impaired wound healing. Artificial dermis has been used for the treatment of full-thickness burns and other areas regarding wound healing. Artificial dermis is composed of an inner collagen sponge layer that stimulates infiltration of the cells and an outer silicon membrane layer that prevents infection. When the artificial dermis is placed on skin defects, fibroblasts, and capillaries infiltrate into the pores of the collagen sponge. Then, the collagen sponge is spontaneously converted into regenerated connective tissue, so-called “dermis-like tissue.” Recently, clinical effectiveness has been reported in combination therapy using artificial dermis and basic fibroblast growth factor. Artificial dermis, proposed by different companies and in particular PELNAC by Gunze is useful for reconstructing various full-thickness skin defect such as third-degree burns, giant nevi, traumas, and donor sites of skin flaps (167). It is also highly useful after resection of malignant skin tumors because it can reduce the sacrificial burden of skin grafts or flaps if extended surgery becomes necessary. Basic fibroblast growth factor (bFGF) is a cytokine that promotes granulation tissue formation and angiogenesis, and when used in combination with PELNAC produced excellent treatment outcomes even for wounds with a high risk of infection (168, 169). Several studies indicate that morbid obesity significantly increased the risk of a post-operative wound complication and infection. Obese women with breast cancer represent a unique patient population. They are at increased risk for the development of breast cancer and may experience more complications related to surgery and medical. Moreover, they may not be candidates for in breast reconstruction due to limited reconstructive options such as wound dehiscence and risk of reoperation following reconstruction (170). However, following surgery obese patients through the use of artificial dermis they should expect a better result without complications. Mendenhall et al. showed that the use of the dermal matrix in breast reconstruction produced an improvement in healing (171). In addition, obesity is the leading risk factor for type 2 diabetes (T2DM), and the risk of T2DM increases with increasing weight classes. Together, obesity and T2DM are risk factors for numerous comorbidities such as cardiovascular disease, gastrointestinal disorders, and osteoarthritis (172). One of the most common complications of diabetes is diabetic foot ulcer (173). Diabetic ulcers do not heal easily due to diabetic neuropathy and reduced blood flow, and non-healing ulcers may progress to gangrene, which necessitates amputation of the patient's foot. In an obese diabetic rat model, Kato et al. have developed a new therapy based on allogenic transplantation of an adipose-derived stem cell sheet combined with artificial skin, and show that it accelerates wound healing (174).

## Conclusion

Excess weight is defined as secondary if it is a consequence of other diseases, such as hypothyroidism or other clearly identifiable causes. In the majority of cases it is instead primary because it is not caused by other diseases, but its development is favored by several factors that interact with each other. Obesity is known to be an important risk factor for cardiovascular disease and diabetes. Less known, but perhaps even more worrying, is the contribution this condition makes to the risk of developing certain cancers, particularly breast cancer. The excess of fat does not exclusively influence the correct and timely diagnosis of breast cancer but, by altering the tumor microenvironment, it can favor its growth, progression, response to therapies, consequently worsening the prognosis. The mechanisms underlying the cancer-obesity association are multiple and distinct for the different types of cancer. Obesity is certainly associated with a series of endocrine and metabolism alterations, in particular the metabolism of sex hormones, insulin, and Igf (insulin growth factor), adipokines (hormones produced by adipose tissue), as well as inflammation. There is strong evidence linking obesity to cancer, through the alteration of the metabolism of sex hormones and chronic inflammation. However, there is clear evidence that weight loss is able to positively influence all these mechanisms, probably by regulating the balance between cell proliferation and apoptosis. Based on the studies available to date, it can be concluded that paying attention to weight reduces the risk of developing most cancers. It seems obvious to talk about many causes at the origin of primary obesity, but still, to most of the overweight or obese subjects, only a diet is prescribed, to reduce the energy introduced every day. This approach is reductive because it must be considered that there are many risk factors of excess weight such as genetic factors, hormonal factors, a sedentary lifestyle, the environment, and eating behaviors that are often influenced by psychological aspects and not least the environment internal: the microbiota. More and more experimental evidence confirms that the microbiota affects body weight. Billions of microbes in the intestine contribute to determining the total weight of the organism, directly influencing the digestion and metabolism of the substrates introduced with the food as well as the quantity of sugars, fats, and proteins absorbed by the intestine. The future lines in the treatment of obesity seem to adopt as new strategies for weight loss, interventions on the microbiota.

## Author Contributions

MD contributed to the draft on breast cancer and obesity topics. FP, LQ, MCon, AS, and GS collected bibliographic data in order to develop the written manuscript regarding oxidative stress. AR, MCop, KF, and AB-F organized a data collection on gut hormones. GN and GF contributed to the wound healing and obesity. MB revised all the manuscript.

### Conflict of Interest Statement

The authors declare that the research was conducted in the absence of any commercial or financial relationships that could be construed as a potential conflict of interest.
