# Erratum: The Role of Oxidative Stress and Hormones in Controlling Obesity

**DOI:** 10.3389/fendo.2019.00693

**Published:** 2019-09-27

**Authors:** Frontiers Production Office

**Affiliations:** Frontiers Media SA, Lausanne, Switzerland

**Keywords:** obesity, oxidative stress, thyroid, gut hormones, microbiota, wound healing

Due to a production error, an author name was misspelled. “Arbereska Bexheti-Ferati” should be “Arbëresha Bexheti-Ferati.”

The publisher apologizes for this mistake. The original article has been updated.

